# Improving reference standards for validation of AI-based radiography

**DOI:** 10.1259/bjr.20210435

**Published:** 2021-06-17

**Authors:** Gavin E Duggan, Joshua J Reicher, Yun Liu, Daniel Tse, Shravya Shetty

**Affiliations:** 1 Google Health (G.E.D., Y.L., D.T., S.S.), Stanford Health Care and Palo Alto Veterans Affairs (J.J.R.), California, California, USA

## Abstract

**Objective:**

Demonstrate the importance of combining multiple readers' opinions, in a context-aware manner, when establishing the reference standard for validation of artificial intelligence (AI) applications for, *e.g.* chest radiographs. By comparing individual readers, majority vote of a panel, and panel-based discussion, we identify methods which maximize interobserver agreement and label reproducibility.

**Methods:**

1100 frontal chest radiographs were evaluated for 6 findings: airspace opacity, cardiomegaly, pulmonary edema, fracture, nodules, and pneumothorax. Each image was reviewed by six radiologists, first individually and then via asynchronous adjudication (web-based discussion) in two panels of three readers to resolve disagreements within each panel. We quantified the reproducibility of each method by measuring interreader agreement.

**Results:**

Panel-based majority vote improved agreement relative to individual readers for all findings. Most disagreements were resolved with two rounds of adjudication, which further improved reproducibility for some findings, particularly reducing misses. Improvements varied across finding categories, with adjudication improving agreement for cardiomegaly, fractures, and pneumothorax.

**Conclusion:**

The likelihood of interreader agreement, even within panels of US board-certified radiologists, must be considered before reads can be used as a reference standard for validation of proposed AI tools. Agreement and, by extension, reproducibility can be improved by applying majority vote, maximum sensitivity, or asynchronous adjudication for different findings, which supports the development of higher quality clinical research.

**Advances in knowledge:**

A panel of three experts is a common technique for establishing reference standards when ground truth is not available for use in AI validation. The manner in which differing opinions are resolved is shown to be important, and has not been previously explored.

## Introduction

The interpretation of radiologic images involves subjectivity owing to variability in human image perception.^1,2^ Even with standardized interpretation guidelines, the interpretation of the most commonly obtained radiologic imaging study, the chest radiograph, demonstrates poor interreader consistency.^3,4^ The causes of variability include inherent limitations of the imaging modality itself, differences in radiologist training, individual subjectivity of readers, and non-standardized guidelines for categorizing findings.^3–6^ Such interreader variability impacts both clinical research and patient care.

Research groups developing deep learning algorithms for radiography^7–12^ have increasingly aimed to achieve independent, “radiologist-level” accuracy; chest imaging has been popular because of the large data sets available. However, the reference standard (also known as ground truth or “label”) used to validate these algorithms needs to be scrutinized as a gold-standard is not available in many clinical situations.^13^ Chest radiograph interpretation, with its inherent limitations, is often the only viable source of reference standard. Inaccuracies in that ground truth invariably lower the quality of the validation, and resulting algorithm.^14^

Typically, reference standards are extracted from clinical radiology reports or reinterpretation of images by one or more readers (where ground truth is defined by the majority of the opinions of the panel).^9^ An alternative method of resolving disparate interpretations in a research setting is adjudication, where readers collaboratively resolve disagreements with or without supplemental clinical data such as pathology or clinical outcomes.^15^ In particular, adjudication, also called “arbitration” or “consensus,” by an expert panel has been posited to be a high-quality reference standard for image interpretation in other modalities.^13^ However, in the absence of data, the inherent issues (*e.g.* bias introduced by vocal leaders and confirmation bias) have been discussed, and alternatives suggested.^16^ One potential alternative is blinded, asynchronous adjudication: multiple rounds of anonymous panel-based discussion via the web to reach agreement on findings.^17^

In this study, we sought to compare the use of multiple methods to maximize label consistency across six important chest radiography findings: airspace opacity, cardiomegaly, pulmonary edema, fracture, nodules, and pneumothorax. We investigated: (1) whether panels of readers via majority vote (or other voting methods) performed more consistently than individual readers; (2) whether blinded, asynchronous adjudication could further improve agreement and what changed with multiple rounds of adjudication; (3) how quickly disagreements within a panel were resolved using this approach; (4) what factors contributed as typical sources of variation within or between panels. Thus, our study extends existing literature on reader variability by quantifying the impact of different methods in producing a consistent reference standard.

## Methods and materials

### Data preparation

This study utilized a retrospective set of deidentified frontal chest radiographs from five regional centers in five cities at a large hospital group in India (Table 1). Institutional Ethics Committee approvals (comparable to Institutional Review Board for institutions in India) for this retrospective study were obtained from all participating institutions.

**Table 1.** Characteristics of images in this study

Data set origin	Five hospitals from five cities in India
Number of patients	1100
Number of images	1100
Age (median, interquartile range)	53 (40–63)
Female (%)	378 (35%)
Image resolution in pixels (median, interquartile range)	Width: 2576 (2500–2902)
	Height: 2365 (2100–2608)

Additional information about the composition of the dataset is available in Supplementary Table 1
Supplementary Table 1. Click here for additional data file. .

Because any given abnormal finding is found in a minority of cases in practice, we enriched the data set for six findings of interest: airspace opacity, cardiomegaly, pulmonary edema, fracture, nodules, and pneumothorax. Findings of fracture and pneumothorax are often complicated by detection challenges (*i.e.* difficult to find); cardiomegaly and edema by thresholding difficulties (*i.e.* subjectivity of whether a detected finding is real and/or significant); and opacity by non-specific classification (*i.e.* ambiguity in what finding the detected opacity represents). Pulmonary nodules are subject to all three problems due to the inherent limitations of chest radiography (*e.g.* compared to CT).

Regarding the identification of pulmonary edema (edema), preliminary data indicated high levels of disagreement between “mild” and “no” edema, associated with the subtle and difficult findings of minimal vascular and interstitial prominence and the prevalence of confounding acquisition artifacts. Given those observations and the greater clinical significance of detecting moderate edema, this study considers findings of “at least moderate edema.”

To enrich the data set, clinical radiology reports containing a variety of keywords associated with each condition were pulled, and a reader not involved in the remainder of this study reviewed the case to confirm the mention of an abnormal finding. This preliminary review of the report was used solely for enrichment, and was independent of the image interpretations in the study. In total, 600 “probable normals” and 500 “probable abnormals” were selected, for a total of 1100 images from 1100 patients. An approximate number of “probable abnormal” cases and the estimated enrichment for each finding are displayed in Supplementary Table 1. Among these images, 434 were also used to train models in a different study involving deep learning.^18^ Labeling for that study’s training data was performed separately by different radiologists, none of whom participated in this study.

### Study design

Our study contained the following stages: initially, each image was independently reviewed by six American Board of Radiology-certified radiologists (“readers”), three from each of two panels (one panel per study arm). Data were then analyzed to evaluate individual reader agreement, inter*panel* agreement using majority vote, and interpanel agreement using “maximum sensitivity” voting; see Statistical Analysis. Finally, the three readers from each panel proceeded through rounds of discussion (“asynchronous adjudication”) to resolve disagreements within the panel. Changes in interpanel agreement across the rounds of discussion were then evaluated.

### Two arms of the study: interpretations by two independent panels

Reviewers consisted of nine radiologists, divided into two cohorts of four and five radiologists, each representing an independent study arm (“arm 1” and “arm 2”). The radiologists in arm 1 and arm 2 had an average of 9 and 6 years of experience, with a range of 7–21 and 3–11 years, respectively. For each arm, a panel comprising three randomly selected radiologists reviewed each image. This resulted in two sets of three interpretations for each image with no overlap between arms.

Image interpretation was carried out at full resolution via a web-based Digital Imaging and Communications in Medicine (DICOM) viewer. Standard picture archiving and communication systems (PACS) tools were available for use, including window/level tool, pan, and zoom. Readers were asked to label each of the six findings as “Present” or “Absent”, with a third “Hedge” option for nodule and pneumothorax. Details such as severity or location of the finding were also gathered.

### Remote asynchronous adjudication within each arm

After the initial labels were provided by each reader independently, each panel separately (without discussions between panels) adjudicated disagreements via a remote and asynchronous iterative process. Specifically, in each round of adjudication, each of the readers (in a random order each round) labeled the image and left comments. These labels and comments about findings were visible to other readers and were used by the readers to discuss and resolve disagreement. To avoid biases, readers were blinded to the identities of the other readers.

**Figure 1.**
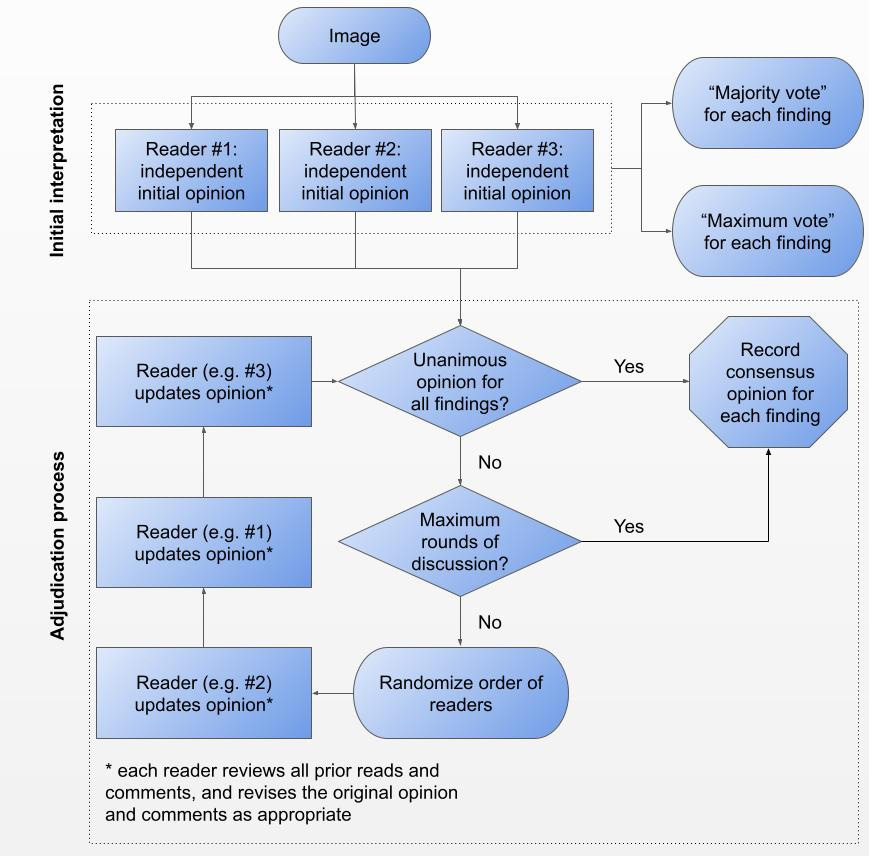
The asynchronous adjudication process used to reconcile differences in the initial three individual opinions (within each panel). Each panel independently followed this process.

In each arm, adjudication proceeded for up to two rounds (Figure 1), stopping early if consensus on both finding presence and additional details (*e.g.* location and severity) was reached on all findings. Including the initial interpretations, a maximum of nine image interpretations across three radiologists were seen in each study arm. Arm 1 further adjudicated any unresolved findings for up to three additional rounds to assess the impact of more rounds of discussion. For the purposes of analysis in this study, any remaining disagreements at the end of the rounds of adjudication was resolved by majority vote. The asynchronous nature of this process enabled readers to label images on a flexible schedule, without the need to align multiple clinical schedules.

### Statistical analysis

#### Summarizing initial interpretations of each arm

Each of the two study arms provided initial, independent, image interpretations from three radiologists chosen at random. To summarize each group’s initial opinions, we compared two “voting” procedures: “majority vote” and “maximum sensitivity.” In the commonly used “majority vote,” a positive finding is reported only if two or three readers indicated its presence. The “maximum sensitivity” voting procedure results in a positive finding if any of the three readers indicated its presence, and is based on the hypothesis that detection problems may be accurately captured by the most sensitive reader for each image (*e.g.* only one reader may have flagged a nodule or subtle fracture).

### Measurement of agreement

In the absence of an unambiguous gold-standard ground truth, our analyses involved measuring agreement between two groups, *e.g.* Arm 1 *vs* Arm 2, or Arm 1 before adjudication *vs* Arm 1 after adjudication. To measure agreement, we calculated Krippendorff’s α (α or ⍺, calculated using the nltk Python package, v. 3.2.2), which handles the variable reader composition for each image’s evaluation better than other agreement metrics.^19^ Values range from 0.0 (indicating a total absence of reliability) to 1.0, indicating perfect reliability/agreement. Notably, α has the benefit of emphasizing agreement in rare findings; though most images were negative for any given finding, even the findings with an α of ~0.3 had an overall concordance exceeding 70%.

For individual reader agreement, α was calculated using a random reader from each arm for each image, to facilitate comparison with aggregated panel opinions on the same number of observations. This was repeated *n* = 100 times and averaged.

Two findings (nodule and pneumothorax) allowed readers to indicate a “Hedge” opinion in place of “Present” or “Absent”. To ensure agreement statistics were comparable across findings, and considering the implications of a hedge on clinical decision-making, α was calculated by treating hedges as “Present” for the purposes of this analysis.

## Results

### Initial interpretations by radiologists

In each of the two arms of our study, 1100 frontal chest radiographs were evaluated for 6 findings (airspace opacity, cardiomegaly, edema, fracture, nodule, and pneumothorax) by 3 board-certified radiologists. The majority vote of the readers in the first arm considered 11 images to be of poor image quality, and these images were excluded from the remainder of the study. Six additional images were excluded due to technical difficulties unrelated to image contents. The agreement (as measured by Krippendorff’s ⍺) between individual readers ranged from ⍺=0.18–0.23 for more subjective findings to ⍺=0.51 for more objective ones (Table 2).

**Table 2.** Agreement as measured by Krippendorff’s α between two individuals, or between two panels of three readers

Finding	Individual reader	Panel			Predominant Sources of Disagreement
		Initial Majority	Maximum Sensitivity^*a*^	Adjudicated Consensus	
Airspace Opacity	0.44	0.62	0.43	0.61	Subjective classification of abnormality
Cardiomegaly	0.51	0.66	0.63	0.68	Subjective thresholding
Edema	0.23	0.30	0.28	0.44	Subjective thresholding
Fractures	0.51	0.64	0.75	0.74	Detection “misses,” particularly subtle fractures
Nodule^*b*^	0.18	0.37	0.28	0.33	Detection, thresholding, and subjective classification
Pneumothorax^*b*^	0.37	0.51	0.55	0.51	Majority vote captures the least ambiguous cases only

Panel opinions were aggregated using one of three methods (majority, maximum, or consensus) before comparison. Regardless of aggregation method, multi reader panels almost always saw better agreement than individuals, with adjudication performing better than majority vote on several findings.

a A panel’s “maximum sensitivity” interpretation of a finding is defined as “Present” if any of the three readers on the panel indicated it is “Present” in the image, contrasting with the majority vote evaluation which requires at least two readers to indicate “Present.”

b Nodule and pneumothorax were sometimes adjudicated as “Hedge.” In this analysis, such images were considered “Present.”

We characterized the reasons for disagreements between readers as differences in detection, thresholding, or classification. Detection differences were related to whether a finding was found and flagged, *e.g.* a subtle fracture. Thresholding referred to subjectivity regarding the presence or significance of an abnormality, *e.g.* whether a subtle density is “real” and could be a small pulmonary nodule. Classification differences are related to the interpretation of a given abnormality, *e.g.* whether a clearly visible opacity represents consolidation associated with infection or alveolar edema. The predominant reasons for disagreement varied by finding, ranging from exclusively detection for fractures to thresholding for edema and classification for airspace opacity (Tables 2 and 3). There were some differences of opinion regarding the clinical relevance of subtle findings (*e.g.* airspace opacity and edema), underscoring the importance of labeling guidelines which specify the desired interpretation for subjective findings.

**Table 3.** Number of disagreements between different categories of findings that are most frequently confused with each other upon initial review by Arm 1, and resolution after discussion

Contention	Adjudicated consensus				
	Opacity	Edema	Both Opacity and Edema	Nodule	None
Edema *vs* nodule	–	1	–	2	–
Opacity *vs* edema	20	11	15	–	5
Opacity *vs* edema *vs* nodule	2	–	2	1	1
Opacity *vs* nodule	4	–	–	2	–

### Comparison of panels’ majority vote (interpanel)

**Figure 2.**
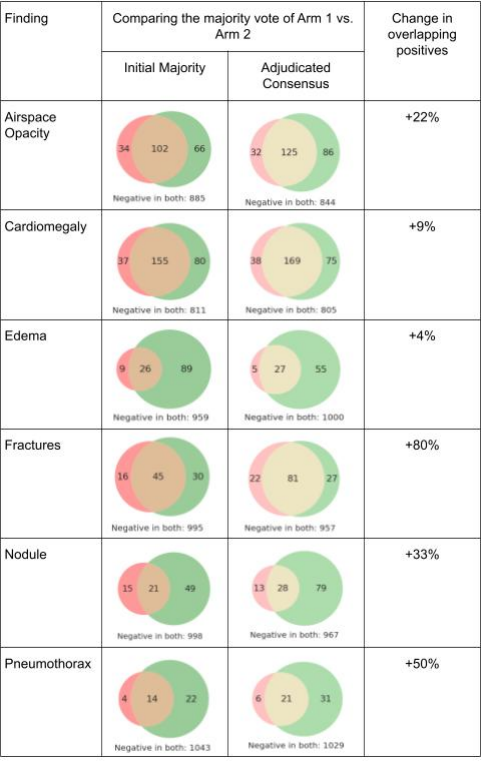
How many images had positive findings in Arm 1 only (red), in Arm 2 only (green) or in both arms (beige). Significant disagreement between panels is seen prior to adjudication (column 2, in darker colors). After adjudication, there are generally more positives (except edema, see decrease in the number of negatives, outside the circles) but also more overlap (column 3, lighter colors).

Next, we assessed the agreement between the majority vote of two panels of readers *prior to* adjudication within each panel. The agreement improved relative to independent reviews for airspace opacity (from ⍺=0.44 to 0.62), cardiomegaly (from ⍺=0.51 to 0.66), and fractures (⍺=0.51 to 0.64), while the agreement for pneumothorax increased but left room for improvement (from ⍺=0.37 to 0.51). Despite improvement, the agreement for edema and nodule remained low at ⍺=0.30 and ⍺=0.37 respectively (Table 2). The number of cases indicated as positive by each arm for each condition is shown as venn diagrams in Figure 2 (column labeled “Before Adjudication”). Interestingly, the maximum sensitivity interpretation of each panel (see Methods) demonstrated similar improvements in agreement for cardiomegaly, edema, and pneumothorax, but no or minimal improvement for airspace opacity and nodule. Remarkably, for fractures the maximum sensitivity opinion of two panels had the highest interpanel agreement at ⍺=0.75 (Table 2).

### Effects of adjudication within each arm (interround)

**Figure 3.**
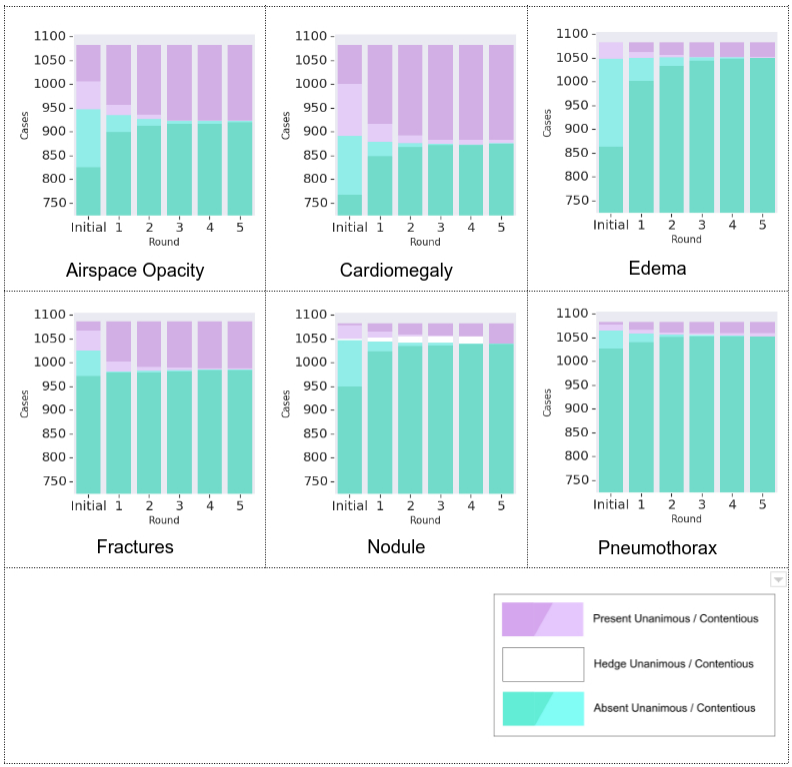
Changes in image interpretation after each additional round of adjudication ￼discussion. It shows the breakdown of the images based on the interpretations by radiologists in arm 1 over additional rounds of discussion. Most changes happened in the first two rounds, number of positives tended to increase (more purple), and adjudication for nodule sometimes ended in hedge (white). Changes in the detailed interpretation of airspace opacity subtype (*e.g.* mass, consolidation, atelectasis) during adjudication are presented in Supplementary Figure 1.

After providing initial opinions, each arm’s panel adjudicated cases with disagreements (number of cases per arm and finding are reported in Supplementary Table 2), with most disagreements (88%) resolving quickly (within two rounds of discussion) across all findings in both study arms (Figure 3). Consistently in both study arms, the number of cases initially interpreted as positive increased after adjudication for all findings except edema, where it decreased (in arm one, *e.g* decrease of 6% for edema, increase of 8–66% for other findings, see Figure 3 for overall numbers).

Supplementary Table 2.

Supplementary Figure 1.

**Figure 4.**
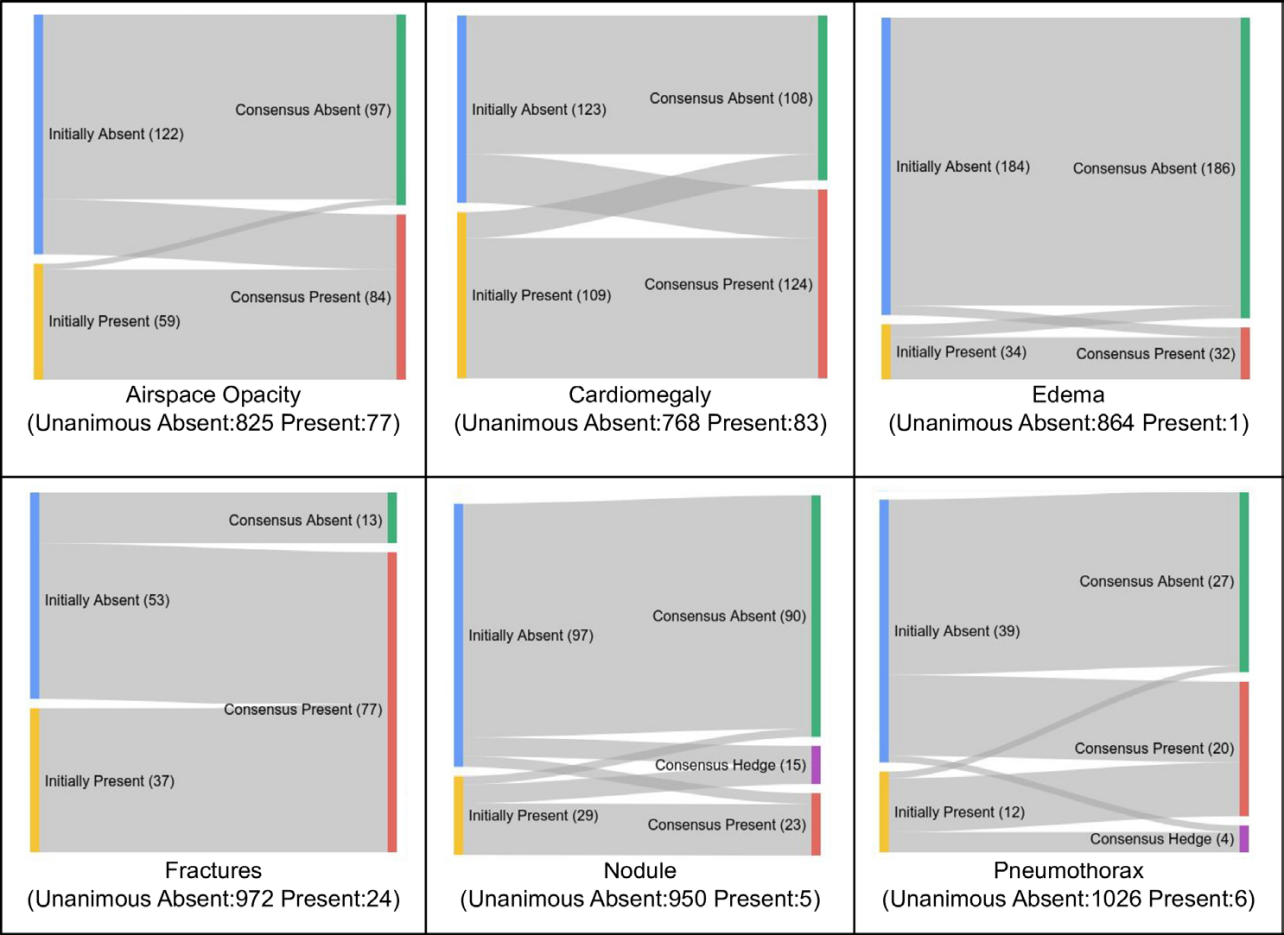
“Flow diagram” illustrating changes in contentious findings (*i.e.* at least one positive and one negative initial read) over the course of adjudication in Arm 1. This is restricted to “contentious” images: those that had disagreement initially (*i.e.* had at least one positive and one negative initial read). Most conditions saw a substantial fraction of both upgrades and downgrades, except fractures which were exclusively marked “Present” during adjudication. For clarity, the nodule plot excludes two cases with an initial majority of “Hedge,” that were adjudicated to “Present” and “Absent,” respectively.

**Figure 5.**
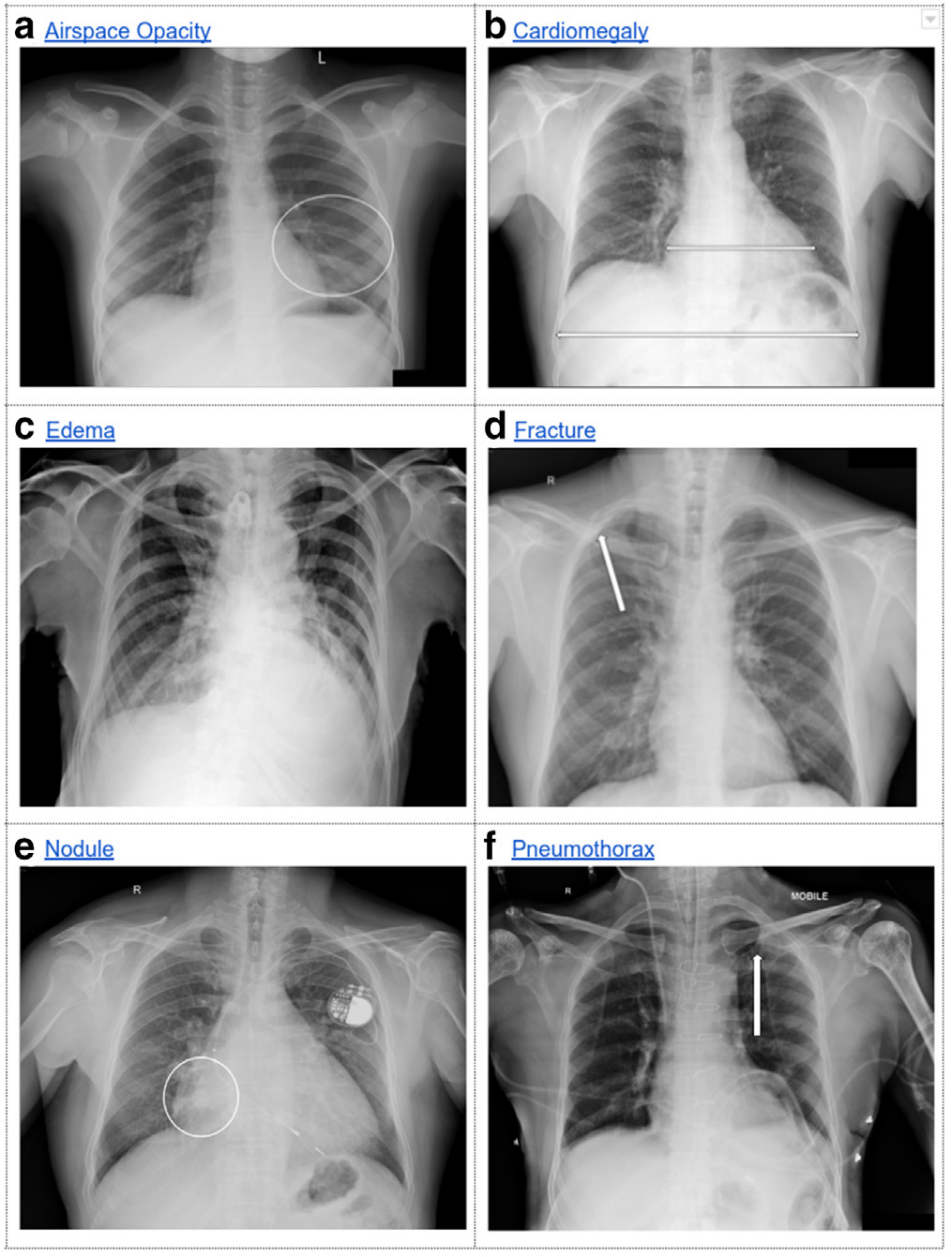
Representative images for the findings studied. (a) Though the majority of readers in arm one initially called this negative for airspace opacity, the disagreement resolved in favor of positive finding of left mid-lung consolidation after further discussion. (b) The majority of arm one initially called this negative for cardiomegaly. However, a cardiothoracic ratio measurement by the dissenting minority reader convinced the other readers. (c) The radiologists in both arms disagreed strongly regarding the presence of edema in this case, resulting in disagreements that did not resolve upon discussion. (d) A fairly representative case for fractures: individual readers missed the right second rib fracture, but quickly accepted once alerted to the finding by another reader. (e) The rounded opacity projecting over the right atrium was initially missed by the majority of readers, and called positive only after multiple rounds of discussion. (f) A pneumothorax was noted at the left lung apex by some readers but not others. With discussion, the two arms resolved this in opposite directions (positive *vs* negative).

Next, we assessed the cause of disagreement resolution, including both cases where the majority called “Present” and those called “Absent” (Figure 4). While both “upgrades” from the majority vote (“Absent” to “Present”) and “downgrades” (“Present to “Absent”) were observed in most findings, fractures almost always resolved to “Present;” if any reader indicated the presence of a fracture, the other readers in a panel generally agreed after adjudication. Edema and nodule demonstrated the opposite trend however; most nodule cases with a majority opinion of “Absent” resolved as “Absent,” though many disagreements (particularly past the first round of discussion) resolved towards “Hedge” (Figures 4 and 5). For edema, most disagreements resolved towards “Absent.”

Airspace opacity also had further subtleties beyond general trends, with two predominant classification issues (Supplementary Figure 1). First, there was disagreement regarding classification of opacity subtype [*e.g.* atelectasis *vs* consolidation (pneumonia)]. Second, there was overlap or coexistence of edema, nodule, and non-nodular opacity in generally ill patients (*e.g.* patients in the intensive care unit with perhaps both pulmonary edema and focal pneumonia). These situations reflect expected and known areas of difficulty in clinical practice, and adjudication resolved disagreements in most cases. For opacity subtype, the most common outcomes were atelectasis, hedge (generally between atelectasis and consolidation), and none (*i.e.* negative for any finding, though potentially with presence of chronic scarring). For the overlap cases, results settled to a mixture of interstitial edema only, opacity only, and coexistence of interstitial edema and opacity (Table 3).

### Comparison between arms after adjudication (interpanel)

**Figure 6.**
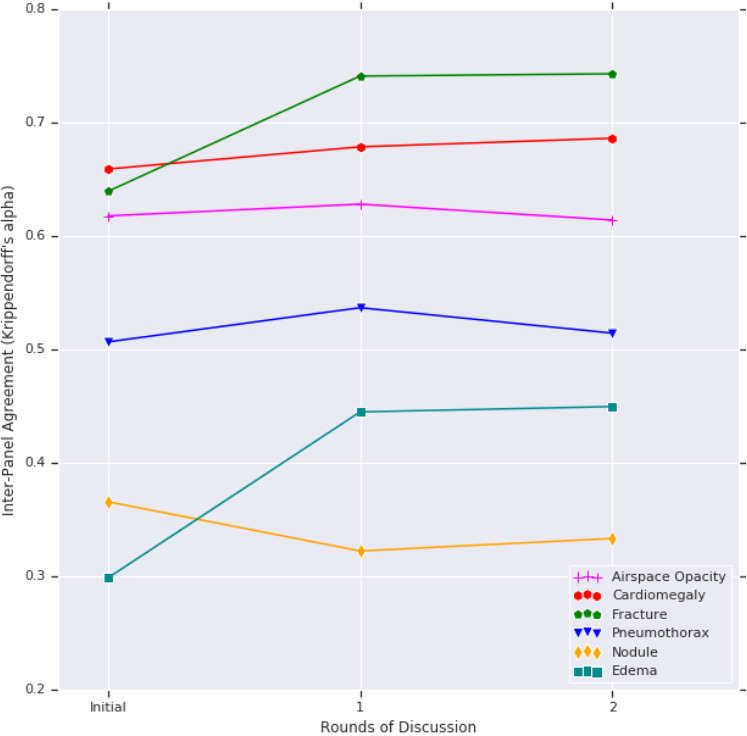
Changes in interpanel agreement (quantified by Krippendorff’s α) with rounds of discussion. For all findings, the largest change occurred in the first round of discussion. For most findings, an improvement in agreement (between arms) was seen after discussion (within each arm), though to different extents depending on the finding. Because the vast majority of images were negative for any given finding, even the findings with an α of ~0.3 have an overall concordance exceeding 70%. See results for an investigation of nodule disagreement between arms.

Finally, we assessed the inter panel agreement between the two study arms, after two rounds of adjudication. Compared to the majority vote of each panel (Table 2), the agreement rose substantially for fractures (from 0.64 to 0.74) and edema (from 0.30 to 0.44). Marginal improvement was seen in the agreement for cardiomegaly (0.66–0.68), while that for airspace opacity and pneumothorax did not change appreciably. The agreement for nodule deteriorated; upon detailed inspection, arm two was consistently more likely to hedge on potential nodules instead of calling them “Absent.” Illustrative example images for each finding are presented in Figure 5, and the trends in inter panel agreement across rounds of discussion are plotted in Figure 6.

To reconcile these results with the observation that the same trend of rapid disagreement resolution and increase in positive findings occurred during adjudication in both study arms, we visualized the overlap in positive findings between study arms (shown as Venn diagrams in Figure 2, column labeled “After Adjudication”). With the exception of fractures, arm two was substantially more sensitive for the remaining five findings, consistently indicating additional positive findings relative to arm 1, with the addition ranging from 25 to 37 cases (cardiomegaly and pneumothorax), to 50–66 (airspace opacity, edema, and nodule). Interestingly, this is despite many positive findings being “removed” during the process of adjudication in arm 2 (*e.g.* 33 out of 116 cases initially positive for edema were adjudicated as negative).

## Discussion

We explored various methods of panel-based adjudication for interpreting chest radiographs, to identify methods which maximize interobserver agreement and “ground truth” quality. Our major findings were: (1) panel-based majority vote improved agreement relative to individual readers; (2) despite being an improvement over individual readers, the majority vote of two panels of radiologists still frequently disagreed on several major findings; (3) the maximum sensitivity voting procedure resulted in high inter panel agreement for some conditions; (4) adjudication resolved many disagreements quickly, typically within 2 rounds of discussion; and (5) adjudication improved the agreement between two panels for most but not all findings studied.

Notably, we augment previous knowledge regarding inter*observer* variability, with data regarding inter *panel* variability: the degree to which the opinions of independent panels agree. These data are suggestive of both the value of adjudication in improving the consistency and reproducibility of reference standards, for multiple findings in chest radiography, and the inherent limitations of subjective image interpretation of chest radiography.

Three main categories of disagreement were observed during adjudication: detection, thresholding, and classification. Detection disagreements describe “misses” occurring during the initial interpretation. Even if only one reader detected the finding (*e.g.* a subtle fracture), adjudication tended to quickly resolve discrepancies (Figure 3, fractures subpanel), suggesting that detection errors can potentially be further reduced with larger panel sizes. Thresholding disagreements described subjectivity in whether an abnormality was “real” or significant, *e.g.* whether “minimal interstitial prominence” should be classified as “normal” or “mild pulmonary edema,” or whether a subtle density is a pulmonary nodule. These disagreements were reduced with adjudication (*e.g.* Figure 3, airspace opacity and cardiomegaly), but tended to persist more than detection disagreements. Such disagreements may be reduced with clear definitions (*i.e.* what specific findings should constitute “mild” *vs* “no” edema). Classification disagreements primarily concerned whether a detected abnormality represented an airspace opacity *vs* edema, and tended to resolve with adjudication (Table 3).

Our asynchronous adjudication methodology via a web-based system highlights another benefit of technology for radiology. The “asynchronous” nature of the system has the practical benefit of avoiding the need to align busy clinical schedules, a task that grows substantially more difficult with larger panels. As a web-based system, geographically separated readers can participate, potentially increasing the diversity of training backgrounds in studies. The use of text-based comments for communication between readers enables the blinding of reader identities, to reduce the “dominant voice in the room” phenomenon that remains a concern in panels comprising different experience levels.^16^ Finally, each review “round” involves a “double read” by that reader because the “round” happens after a period of time instead of immediately during the same session. Although the reader’s previous grades and comments are visible, the reader has the opportunity to reevaluate the image, though at the cost of the additional read’s time.

Multiple complex issues can lead to variability in chest radiograph image interpretation,^20,21^ including inherent limitations in the modality, including noise, limited spatial resolution, and low contrast resolution (limiting contrast between different body tissues), as well as anatomic overlap and pathology overlap (*e.g.* infection or tumor may look identical).^22^ Interobserver variability is also driven by differences in training, operating points (*i.e.* inherent sensitivity and specificity thresholds), and skill. Lastly, while our work does not address this topic, radiologists are trained to interpret images in varied clinical contexts, which directly impacts selected outcomes and the inherent value of the imaging test itself.^23,24^

The impact of interobserver variability spans both clinical decision-making and clinical research. In clinical practice, double-reading has been used variably to achieve greater consistency in interpretation accuracy.^25^ In research, developing and validating deep learning algorithms in chest radiography often relies on majority vote or even single readers for algorithm validation.^10^ As applications of deep learning in medical imaging proliferate, their rigorous evaluation is becoming increasingly critical. Our research strongly suggests that algorithm validation should utilize higher-reliability ground truth than typically used, via the consensus of multiple experts. Individual reader opinions may be particularly inadequate for “hard to find” findings (which are often of great importance.

For chest radiography specifically, we suggest the following approaches for determining imaging ground truth when clinical outcomes are impractical to obtain. For detection tasks such as fractures (or to a lesser extent pneumothorax), where a false negative may have substantial and immediate clinical consequences, using the “maximum sensitivity” voting procedure of the initial opinions results in remarkably high interpanel agreement (Table 2). For tasks with substantial variance based on thresholds (*e.g.* edema and cardiomegaly), agreed-upon thresholds should be carefully clarified, and panel consensus may be most appropriate. For tasks with classification subjectivity (*e.g.* airspace opacity subtyping and calling nodules), the majority vote of a panel may suffice but additional data from another imaging modality may simply be needed.

The speed of disagreement resolution observed in our study further suggests that panel-based asynchronous adjudication is quite tractable as a solution for research studies, where the speed/accuracy tradeoff is different from clinical workflows.

This study has several limitations. First, the readers participating in this study were board-certified but not fellowship trained. Second, by focusing solely on image interpretation, this study was able to remove the effects of clinical priors and biases introduced by the availability of additional clinical information. On the flip side, confirmation of findings based on cross-sectional imaging was not available.

In conclusion, we studied the variability between panels to gain insights into methodologies for building more reliable and consistent “ground truth” labels for chest radiographs. Combining different techniques, based on the nature of the finding, maximizes agreement and supports development of higher quality reference standards.
