# Supplementary material for: Improving reference standards for validation of AI-based radiography
Source: Br J Radiol. 2021 Jun 17;94(1123):20210435. doi: 10.1259/bjr.20210435 (PMC8248225; doi:10.1259/bjr.20210435)
Supplement: Supplementary Table 1. [file bjr.20210435.suppl-01.docx]

# Supplemental Materials

| **Finding** | **Suspected Positive Images** | **Sample prevalence** | **Approximate Enrichment** |
| --- | --- | --- | --- |
| Airspace Opacity | 50 | 4.5% | - |
| Cardiomegaly | 100 | 9% | < 3X |
| Edema | 100 | 9% | < 2X |
| Fractures | 50 | 4.5% | - |
| Nodule/Mass | 40 | 3.5% | 5X |
| Pneumothorax | 60 | 5.5% | 10X |
| No finding |  | 60% |  |

**Supplemental Table 1:** Number of images in the evaluation dataset which contained a “prior suspected positive” for each finding (based on keyword search of radiology reports). The enrichment used for each finding was a balance between expected population prevalence, labeling cost, and the need for experimental power.

| Arm | Airspace Opacity | Cardiomegaly | Edema | Fracture | Nodule | Pneumothorax |
| --- | --- | --- | --- | --- | --- | --- |
| 1 | 181 | 232 | 218 | 95 | 128 | 51 |
| 2 | 365 | 302 | 559 | 112 | 249 | 90 |

**Supplemental Table 2**: Number of images required adjudication (i.e. did not achieve consensus on the initial read) for each finding. Arm 2 saw much more disagreement in initial reads.


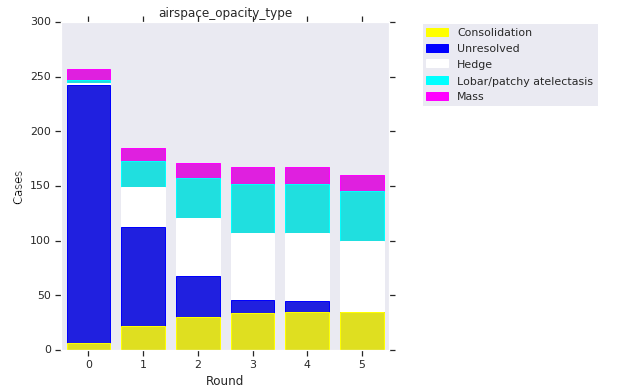


**Supplemental Figure 1**: Resolution of the airspace opacity finding across rounds of discussion in arm 1. The overall count goes down as consensus for some images moved to “Absent” (No opacity).
